# Involvement of Lhcb6 and Lhcb5 in Photosynthesis Regulation in *Physcomitrella patens* Response to Abiotic Stress

**DOI:** 10.3390/ijms20153665

**Published:** 2019-07-26

**Authors:** Xingji Peng, Xingguang Deng, Xiaoya Tang, Tinghong Tan, Dawei Zhang, Baohui Liu, Honghui Lin

**Affiliations:** 1 Ministry of Education Key Laboratory for Bio-Resource and Eco-Environment, College of Life Science, State Key Laboratory of Hydraulics and Mountain River Engineering, Sichuan University, Chengdu 610064, China; 2 School of Life Sciences, Guangzhou University, Guangzhou 510006, China

**Keywords:** minor antenna protein, *Physcomitrella patens*, growth malformation, chloroplast ultrastructure, photoprotection

## Abstract

There are a number of highly conserved photosystem II light-harvesting antenna proteins in moss whose functions are unclear. Here, we investigated the involvement of chlorophyll-binding proteins, *Lhcb6* and *Lhcb5*, in light-harvesting and photosynthesis regulation in *Physcomitrella patens*. *Lhcb6* or *Lhcb5* knock-out resulted in a disordered thylakoid arrangement, a decrease in the number of grana membranes, and an increase in the number of starch granule. The absence of *Lhcb6* or *Lhcb5* did not noticeably alter the electron transport rates. However, the non-photochemical quenching activity in the *lhcb5* mutant was dramatically reduced when compared to wild-type or *lhcb6* plants under abiotic stress. *Lhcb5* plants were more sensitive to photo-inhibition, while *lhcb6* plants showed little difference compared to the wild-type plants under high-light stress. Moreover, both mutants showed a growth malformation phenotype with reduced chlorophyll content in the gametophyte. These results suggested that *Lhcb6* or *Lhcb5* played a unique role in plant development, thylakoid organization, and photoprotection of PSII in *Physcomitrella*, especially when exposed to high light or osmotic environments.

## 1. Introduction

To harvest solar energy efficiently, photosynthetic organisms use groups of light-harvesting antenna proteins that bind carotenoids and chlorophylls. In higher plants, these proteins are composed of an inner and outer antenna. The photosystem (PS) II outer antenna consists of different heterotrimers of Lhcb1, Lhcb2, and Lhcb3, which is encoded by *Lhcb1*, *Lhcb2,* and *Lhcb3* genes, while minor antenna complexes, such as chlorophyll protein 29 (CP29), CP26, and CP24 are encoded by *Lhcb4*, *Lhcb5,* and *Lhcb6*, respectively [1]. A structural analysis of an Lhcb super-complex and PSII organization has revealed that Lhcb5 (also named CP26) is located between the strongly bound LHCII trimer and dimeric PSII core complex, whereas Lhcb4 (also named CP29) is situated on the opposite side of the dimeric PSII core complex which is associated with moderately bound LHCII trimer and Lhcb6 (also named CP24) [2]. Additional LHCII trimers surround the PSII structure by connecting with Lhcb6 to the core of PSII in higher plants [3,4].

There are two mechanisms that regulate the absorption of light energy from Lhc (light-harvesting complex): (1) the light-harvesting antennas quench excess harvested light, which is known as non-photochemical quenching (NPQ) [5,6]; (2) state transition, which is a process associated with PSII or PSI that can balance the electron transfer [7]. The largest NPQ is dependent on a low thylakoid lumen pH and defined as energy quenching (qE) [8]. Among these minor antenna proteins, only *Lhcb4* can be phosphorylated in monocotyledons, and this phosphorylation is involved in state transitions and photo-inhibition recovery [9,10]. The photo-protective role of CP29 phosphorylation reduces singlet oxygen production and enhances excess energy dissipation [11]. Other minor antennas, such as Lhcb6 and Lhcb5, participate in the xanthophyll cycle, which can alleviate light damage [12,13,14,15].

The moss, *Physcomitrella patens,* represents an excellent system to study gene function due to its high homologous recombination frequency and complete sequence genome [16]. Research on the organism has diverged from green lineages to vascular plants and enriches our understanding of how photosynthetic organisms adapt to different environmental conditions [17]. The PSI protein super-complex component Lhcb9 was reported to change the absorption properties of PSII by harboring red-shifted chlorophyll [18,19]. *Physcomitrella* contains both LhcSR (light-harvesting complex stress-related) and PsbS (PSII subunit S) [20]. LhcSR is an ancient light-harvesting protein that has been reported to regulate excess light energy absorption and dissipation in green algae, while in higher plants, LhcSR is functionally replaced by PsbS proteins [21]. However, little is known about the minor antenna proteins in *Physcomitrella*.

In this work, our results showed the function of minor antenna proteins *Lhcb6* and *Lhcb5* in light-harvesting and regulation of photosynthesis when exposed to environmental stress conditions by generating specific mutants that lacked one or both proteins. Under conditions of high light or osmotic stress, NPQ was dramatically reduced in mutants. Moreover, mutants showed deformed leaves, a lower content of chlorophyll content and PSII activity, which suggests that *Lhcb6* and *Lhcb5* played important roles in the organization of photosynthetic complexes in grana partitions. Taken together, our results indicated that minor antenna protein Lhcb5 and Lhcb6 of *Physcomitrella* played significant roles in the function and structure of PSII, especially under abiotic stress.

## 2. Results

### 2.1. Knock-Out of Lhcb6 or Lhcb5 Altered Chloroplast Organization and Lhc Proteins Accumulation

**Figure 1:**
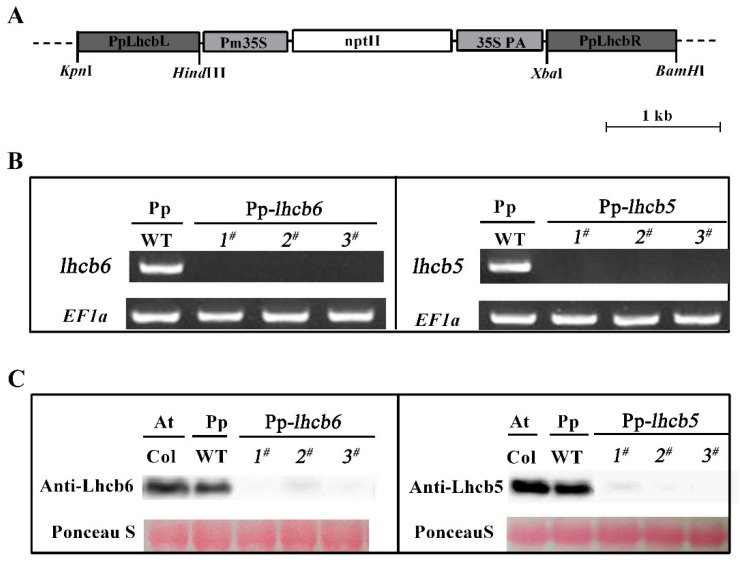
The identification of knock-out mutants. (**A**) The plasmid vector’s schematic maps of pTN182-*PpLhcb5/6* used for knock-out a generation. (**B**) The expression of *PpLhcb6* and *PpLhcb5,* in wild-type and knock-out mutants, was analyzed by RT-PCR. (**C**) Immunoblotting of Lhcb6 and Lhcb5 from *Arabidopsis* (Col) and *Physcomitrella* (wild-type (WT) and lines of knock-out mutants). The chlorophyll content of every lane was 15 μg. Proteins were stained with Ponceau S and Rubisco proteins were used as loading controls.

**Figure 2:**
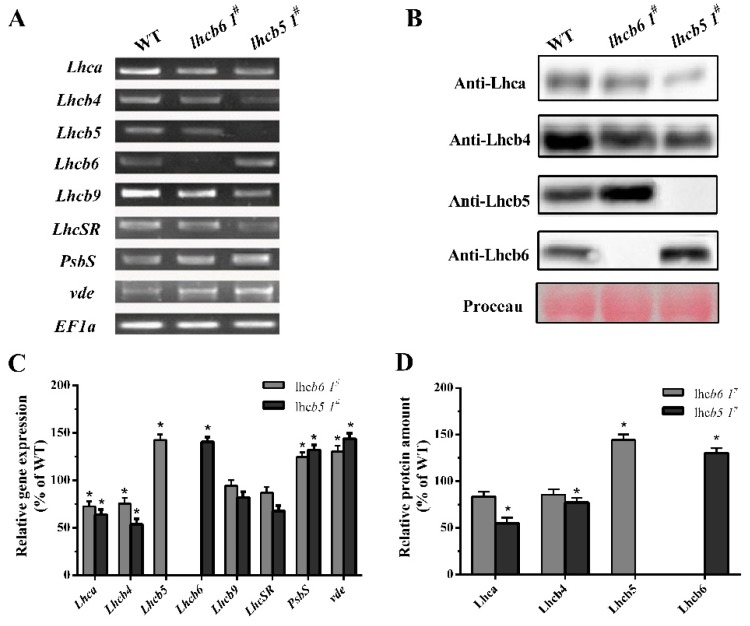
Analysis of *Lhc* genes or protein accumulation in wild-type and mutants. (**A**) The expression of *Lhc*-related genes was analyzed by RT-PCR. *EF1a* was used as reference gene. *Lhc*, Light-harvesting complexes. *PsbS*, PSII subunit S. *vde*, violaxanthin de-epoxidase. (**B**) Immunoblotting of the Lhca, Lhcb4, Lhcb5 and Lhcb6 in wild-type and mutants. Rubisco proteins were used as loading controls and were stained by Ponceau S. (**C**) Quantification of *Lhc* expression levels in WT and mutants. (**D**) Immunological quantification of Lhc proteins in thylakoid membranes. The data represent means ± SD of three biological replicates. Statistical significance compared with the wild-type p is indicated by asterisks (** *p* ≤ 0.01, * *p* ≤ 0.05, Student’s *t*-test).

Although the mutations of *Lhcb6* and *Lhcb5* in higher plants inhibit the interaction of photosystem II subunits and electron transport rate in grana membranes [22], little is known about the effects of minor antenna proteins in *P. patens*. To investigate the physiological function of minor antenna proteins, we generated *Lhcb6* or *Lhcb5* knock-out mutants in *Physcomitrella*. At least three independent lines were isolated and retained for further characterization. The expression of the gene level and accumulation of protein level were detected in mutants by qRT-PCR and Western blotting. As shown Figure 1B,C, mRNA and protein levels were hardly detected in the corresponding mutants when compared with wild-type. The alterations in Lhc stoichiometry in the thylakoid of mutants were verified by immunoblotting and quantitative PCR analysis (Figure 2). In *lhcb6 1^#^*, the expression levels of Lhc components *Lhca* and *Lhcb4* were decreased sharply, while *Lhcb5*, *PsbS* and *violaxanthin de-epoxidase* (*vde*) were increased compared with wild-type plants. Apart from induced *Lhcb6*, all these genes in *lhcb5 1^#^* displayed the same expression pattern as *lhcb6 1^#^*. In addition, the *LhcSR* subunit content did not show obvious changes in *lhcb6 1^#^*, but demonstrated an evident decline in *lhcb5 1^#^*. The *LhcSR* subunit content was reduced in *lhcb5 1^#^* but only changed by a small amount in *lhcb6 1^#^*.

**Figure 3:**
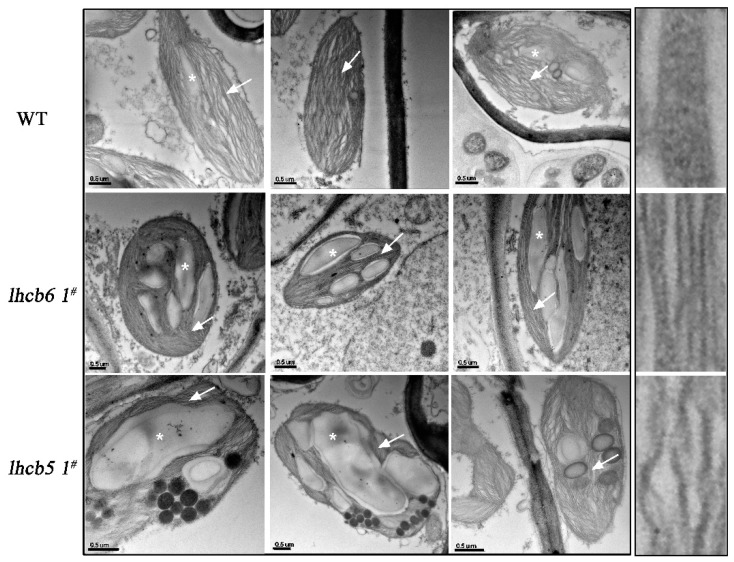
Transmission electron micrograph of plastids from mesophyll cells in wild-type and mutants. Starch granules marked with asterisks can be distinguished from plastoglobules in black dots. The lamella of thylakoid stack and grana are indicated by an arrow.

To investigate the effects of *Lhcb6* and *Lhcb5* on chloroplast organization, ultrathin sections of leaves were analyzed (Figure 3). Under normal growth conditions, wild-type (WT) plant chloroplasts showed a characteristic organization of stromal membrane with interconnecting grana stacks and large starch granules in most sections. The *lhcb6 1^#^* plants showed a lamella with a disordered arrangement of thylakoids, reduced stacked grana, and increased plastoglobules and starch granules. Chloroplasts from mutant *lhcb5 1^#^* accumulated more starch granules and plastoglobules but displayed a higher ratio of stromal membrane to grana stacks when compared with WT. These results demonstrated that Lhcb6 and Lhcb5 influenced the ultrastructure of chloroplast and the accumulation of thylakoid proteins.

### 2.2. PSII Activity is Markedly Reduced in Mutants under High-Light Treatment

**Figure 4:**
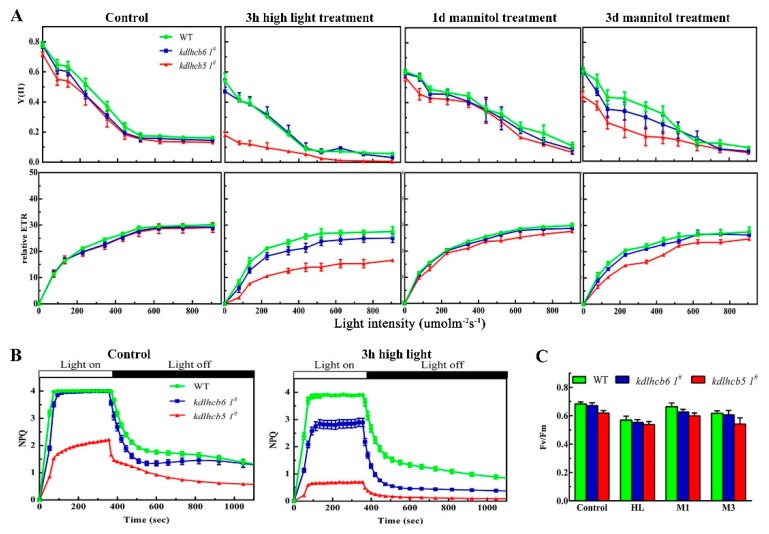
Detailed characterization of chlorophyll fluorescence in wild-type and mutants. (**A**) Light-response curves of PSII quantum yield and ETR in wild-type and mutants. (**B**) Time courses for induction and relaxation of non-photochemical quenching (NPQ) before, and after, 3 h of high light treatment. (**C**) The maximal photochemical efficiency of PSII. HL represents high light treatment for 3 h. M1 or M3 represent plants under 500 mM mannitol for 1 day or 3 days, respectively. The data represent the means ± SD of three biological replicates. Statistical significance compared with the wild-type p is indicated by asterisks (* *p* ≤ 0.05, Student’s *t*-test).

As the chloroplast organization was altered in mutants, we further analyzed PSII function in different mutants using fluorescence measurements. The peaks in the room temperature fluorescence emission spectra of mutants were lower when compared with wild-type at 685 nm (Supplementary Figure S1), suggesting that Lhcb6 and Lhcb5 affected the fluorescence emission spectra of several chlorophylls and the light-harvesting efficiency of PSII. To understand the primary functions of Lhcb6 and Lhcb5 in *P. patens*, non-invasive chlorophyll fluorometric analyses were performed to investigate the photosynthetic electron transport in wild-type and mutants (Figure 4, Supplementary Figure S3). The initial Fv/Fm measurements demonstrated that PSII activity was disturbed in mutants and wild-type after high-light treatment (Figure 4A). To further investigate the PSII activity of mutants under abiotic stress, the maximum (Fm) and minimum (F_0_) fluorescence, and non-photochemical quenching (NPQ) of dark-adapted plants were quantitatively determined. Under normal growth conditions, *lhcb5 1^#^* showed significantly decreased NPQ compared to wild-type (Figure 4C).

The NPQ amplitude has been reported to be dependent on the lumen pH or on the concentration of PsbS [13,23]. Proton pumping into the chloroplast lumen was influenced in *lhcb5 1^#^* rather than *lhcb6 1^#^* and wild-type (Supplementary Figure S2). This result is consistent with the hypothesis that the limitation of NPQ in *lhcb5 1^#^* is partly associated with reduced acidification of the lumen under illumination. The impact of the loss of *Lhcb6* or *Lhcb5* on photosynthesis was investigated by measuring the electron transport activity (ETA) in CO_2_-saturating conditions. The electron transport activity (ETA) in CO_2_ saturating conditions was lower in mutants compared with that in wild-type plants (Supplementary Figure S2B). To further characterize the photosynthetic apparatus, the light-response curves of PSII quantum yield (Y(II)) and electron transport rate (ETR) were also analyzed. After 3 h of high-light treatment, Y(II) and ETR in *lhcb5 1^#^* were significantly reduced compared with wild-type plants (Figure 4A). These results indicated that the minor antenna protein *Lhcb5* was involved in excitation energy via non-photochemical pathways.

### 2.3. PSII Complexes Accumulation Were Affected after High-Light Treatment

**Figure 5:**
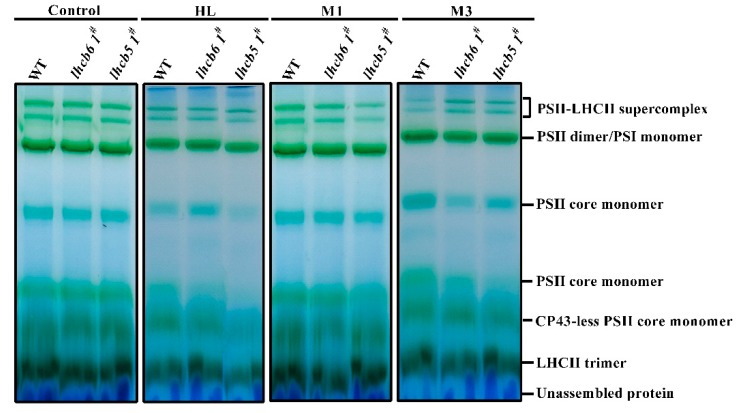
Analysis of pigment-protein complexes in wild-type and mutants by BN-PAGE gel. A freshly isolated thylakoid membrane of wild-type and mutants were solubilized with 1% DM at a chlorophyll concentration of 25 μg. HL means high light stress for 3 h. M1 or M3 means plants under 500 mM mannitol for 1 day or 3 days, respectively. Assignments of the thylakoid membrane macromolecular protein complexes, indicated on the right, were identified according to a previous study [25].

After a short-term high-light treatment, the proteins of the PSII reaction center were damaged. Then, a rapid repair and reassembly process occurred to enable photosynthetic electron transportation [24]. The defected photosynthesis in mutants was possibly caused by a reduced level of protein complexes in the electron transport chain. To further investigate the effects of Lhcb6 and Lhcb5 on the formation of thylakoid membrane protein complexes under abiotic stress, thylakoid membranes (with equal amounts of chlorophyll) in different plants were solubilized in 1% n-dodecyl-β-D-maltoside (DM) and the chlorophyll–protein complexes were separated by BN-PAGE. Six protein complexes, PSII-LHCII super-complex, PSII dimer/PSI monomer, PSII core monomer, CP43-less PSII core monomer, LHCII trimer, and unassembled protein were resolved (Figure 5). Under high-light stress, the PSII core monomer of the *lhcb5 1^#^* mutant was reduced, suggesting Lhcb5 might associate with the formation and stability of the PSII complex. Under osmotic stress, the amount of monomer and free pigment of mutants did not show significant differences compared with wild-type plants. These results suggest that light was a critical abiotic factor that limits the photosynthesis efficiency of PSII in *Physcomitrella*.

### 2.4. Growth Malformation Phenotype of Mutants

**Figure 6:**
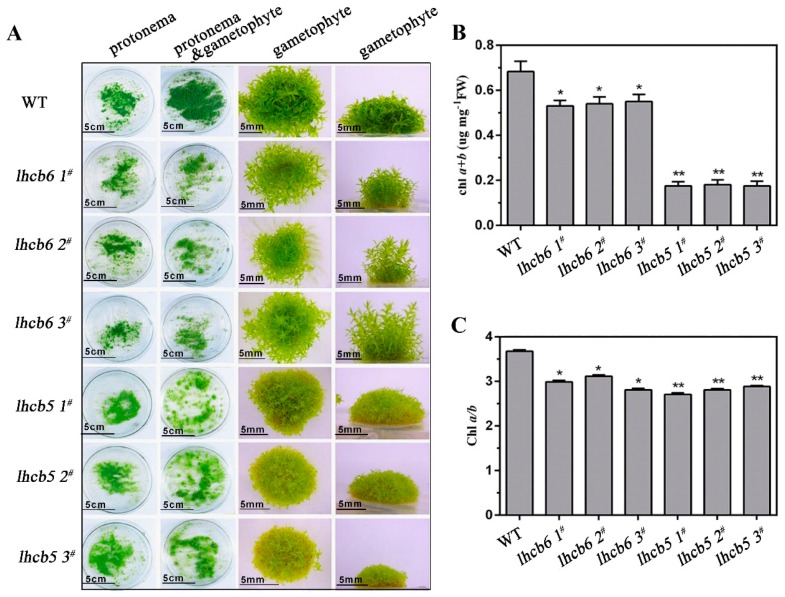
The phenotype and chlorophyll content of wild-type and mutant plants. (**A**) The protonema and gametophyte phenotypes of wild-type and mutants. The protonema tissues of wild-type and mutants were grown for one week on ammonium tartrate-free BCD medium. The protonema and gametophyte tissues were grown in this medium for four weeks. The gametophyte tissues were grown in the same medium for six-weeks. The chlorophyll *a+b* content (**B**) and chlorophyll a/b ratio (**C**) of leafy gametophyte tissues. FW, Fresh weight. The data represent the means ± SD of three biological replicates. Statistical significance compared with the wild-type is indicated by asterisks (** *p* ≤ 0.01, * *p* ≤ 0.05, Student’s *t*-test).

Even though there was no significant difference in the protonemal filaments between mutants and wild-type plants, the leafy gametophyte showed large differences. The leafy gametophyte of *lhcb6* plants grew slender and higher, with relatively narrower and longer leaves, while *lhcb5* showed a more serious malformation phenotype when compared with *lhcb6* or wild-type plants (Figure 6A, Supplementary Figure S4A). In addition, the absence of Lhcb5 or Lhcb6 resulted in reduced lateral buds and chloroplast cells in protonema (Supplementary Figure S7). In addition, both mutants, especially the *lhcb5* plants, displayed a dramatically reduced chlorophyll *a+b* content and a noticeably lower chlorophyll *a/b* ratio in comparison with wild-type plants (Figure 6B,C, Supplementary Figure S4B,C).

In summary, the *Lhcb5* or *Lhcb6* deletion mutant showed severe growth inhibition and developmental deficiency. The remarkable growth inhibition in different mutants suggested that *Lhcb6* and *Lhcb5* played essential roles in leaf development of *Physcomitrella*.

## 3. Discussion

Light-harvesting complexes (Lhc) are members of a large multigene family. They play important roles in regulating photosynthesis in plants’ response to environmental stress. The function of minor chlorophyll-binding proteins is in bridging major LHCII antenna to a dimeric PSII core complex [22,23,26]. Both Lhcb6 and Lhcb5 have a single energy transfer process from Chl b to Chl a [27]. Structural analysis of the Lhcb supercomplex and PSII organization has revealed that Lhcb5 is located between the strongly bound LHCII trimer and dimeric PSII core complex, whereas Lhcb6 is always associated with the connection of LHCII trimers and the core of PSII in higher plants [2,3,4,28]. The *Physcomitrella* has demonstrated a capacity for Lhc-dependent mechanisms in response to environmental conditions in previous research [17,29,30].

To investigate the functions of Lhcb6 and Lhcb5 in *Physcomitrella*, the *PpLhcb6* and *PpLhcb5* were inactivated by a targeted gene replacement assay. The mRNA or protein levels of *Lhcb6* and *Lhcb5* were undetectable in three independent mutation lines (Figure 1). Moreover, the expression of *Lhc* components in both mutants were significantly changed (Figure 2). Interestingly, the *lhcb5* 1^#^ showed an obvious increase in *Lhcb6*, *PsbS* and *vde* accumulation, in addition to decreased *Lhca* and *Lhcb4* levels. The *lhcb6* 1^#^ mutant showed similar expression levels of pigment-protein components. Under high-light stress, the efficiency of excitation energy trapping and non-photochemical quenching in the absence of the *lhcb5* mutant were much lower than WT plant, while the *Lhcb6* deletion mutant only displayed a dramatic decrease in non-photochemical quenching (Figure 4). PsbS and LhcSR regulate excess energy dissipation by the xanthophyll cycle, which is an important anticipatory strategy against photo-inhibition [31]. Lhc proteins are associated with scavenging of reactive oxygen speciecs (ROS) species by switching back and forth between PSI and PSII. The direct quenching of chlorophyll triplet states by xanthophyll is associated with VDE and LhcSR [12,30,32]. The mechanisms which balance light absorption and the amount of electron transport acceptor substrates clarify the evolution of oxygenic photosynthesis [31]. Here, the different expression levels of *Lhc* genes observed in different mutants might be interpreted as compensatory mechanisms aimed at the dissipation of excess energy.

In higher plants, the minor antenna proteins Lhcb6 and Lhcb5 affect the interactions of PSII subunits and the electron transport rate in grana membranes, especially for limiting plastoquinone diffusion [22,23,26]. Here, the absence of either *Lhcb5* or *Lhcb6* influenced the ultrastructure of chloroplast and the accumulation of thylakoid proteins, and particularly, reduced the number of stacked grana and increased the amount of starch granules (Figure 3). The disordered arrangement of thylakoid lamella and increased starch granules suggested that Lhcb6 and Lhcb5 might maintain the chloroplast ultrastructure in vivo.

The structural analysis of the Lhc supercomplex and PSII organization revealed that Lhcb5 is located between the strongly bound LHCII trimer and dimeric PSII core complex, while Lhcb6 is associated with the connection of LHCII trimers and the core of PSII in higher plants [2,3,4,28]. The regulation of light-harvesting and energy transfer from LHCII to the PSII core does not require Lhcb5 [3,23]. In the absence of *Lhcb5*, the NPQ and Y(II) of chlorophyll fluorescence after 3 h of high-light treatment were lower than the plants lacking *Lhcb6* (Figure 4, Supplementary Figure S6). After 3 h of high-light treatment or 3 d of osmotic treatment, *lhcb5 1^#^* showed significantly reduced PSII core monomer protein levels in comparison to WT (Figure 5). Under high-light stress conditions, the *Lhcb5* deletion mutant showed reduced PSII core monomer protein levels, and decreased levels of PSII quantum yield, electron transport rate and NPQ, suggesting that the interaction between LHCII and the PSII super-complex was altered. These results implied the function of the minor antenna protein, *Lhcb5*, might be associated with the formation and stability of PSII super-complex assembly in the thylakoid membrane.

The results described above demonstrated that Lhcb6 and Lhcb5 participated in the accumulation of thylakoid proteins. These two minor antenna proteins probably interacted transiently with PSII complex or some PSII subunits, in a manner similar to several PSII auxiliary factors that interacted with PSII subunits. The cooperative interaction between the PSII core and minor Lhcbs is disrupted, which leads to a decreased affinity when Lhcb6 or Lhcb5 is in low amounts [22]. Furthermore, the inactivation of Lhcb6 or Lhcb5 displayed pleiotropic effects on photosynthetic electron transport. Chlorophyll fluorescence measurements showed that Fv/Fm was slightly reduced in mutants, suggesting no impairment of the PSII complexes (Figure 4), which is similar to previous evidence [33]. A dramatic decrease in NPQ indicated that the photoprotection of PSII was severely inhibited in mutants. These results demonstrated that minor antenna proteins *Lhcb6* and *Lhcb5* affected the chloroplast ultrastructure, the accumulation of PSII complexes and PSII activity under abiotic stress conditions.

Under different abiotic stress conditions, chloroplasts, peroxisomes, and mitochondria accumulated a large amount of reactive oxygen species, which led to cell death and cytotoxicity. Multiple Lhc isoforms assemble with supramolecular photosynthetic complexes to dissipate excess absorbed energy and scavenging ROS. Under high-light stress, Lhc components of the PSII antenna system use non-photochemical quenching to limit the over-reduction of the electron transport chain and the formation of toxic ROS [34,35,36,37]. PsbS and LhcSR regulate the excess energy dissipation by the xanthophyll cycle, which is an important anticipatory strategy in the fight against photo-inhibition [31]. Lhc proteins associate with ROS scavenging by switching back and forth between PSI and PSII, and the direct quenching of chlorophyll triplet states by xanthophyll is associated with VDE and LhcSR [12,30,32]. Compared with the vascular plants, a stronger NPQ and higher capacity of dissipating excitation energy as heat were observed in mosses [17,20,30]. In *Physcomitrella*, it has been shown that NPQ requires LhcsR and PsbS to regulate excess light energy absorption and dissipate light damage [17,20,30,38]. NPQ is also viewed as a compensatory mechanism in response to overexcited chlorophyll to prevent irreversible damage to PSII [39]. In our study, the mutants showed clear differences in the Lhcb polypeptide composition, especially in PsbS and VDE. The expression of *PsbS* and *vde* were increased, while *Lhca*, *Lhcb4,* and *LhcSR* were decreased in the *Lhcb5*-defective mutant. The alteration of chloroplast organization and photosynthesis efficiency suggested that the absence of *Lhcb5* might alter the structure or stability of PSII super-complexes (Figure 3). NPQ activation, in response to illumination with strong actinic light- of dark-adapted plants, was lower in *lhcb5 1^#^*, especially under high-light stress (Figure 4). The distinctly decreased NPQ suggested that the absence of *Lhcb5* resulted in the obstruction of excess light energy transfer between LhcII trimer and PSII core complexes. PsbS and xanthophyll control the affinity of different LhcII antenna complexes for protons to participate in the feed-back control of excess light energy that underlies non-photochemical chlorophyll fluorescence quenching [40]. Although the individual antenna components were not quantified in the analyses on the accumulation of PSII complexes, the chlorophyll *a+b* contents and chlorophyll *a/b* ratios of mutants suggested there may be some minor changes in the antenna in response to high-light treatment. Similarly, the distinguishable electron transport reflected the lower rate of photosynthetic electron transport in mutants (Supplementary Figure S2). An alternative explanation might reflect the role of Lhcb6 and Lhcb5 in channeling prtons away from PSII due to different pH levels in these plants [3,22]. Partial electron transport reactions localized at the restricted step between the plastoquinone site of PSII and the cytochrome *b_6_f* complex for electron donors to cytochrome *b_6_f* are effective in sustaining NADP^+^ reduction [22]. A lack of Lhc proteins might result in the restriction of PQH diffusion from the PSII Q_B_ site to the cytochrome *b*_6_*f* complex.

In our findings, the absence of *Lhcb5* or *Lhcb6* resulted in similar growth-defect phenotypes, such as reduced lateral buds and chloroplast cells in protonema (chloronema and caulonema) and thinner leaves in mature plants (Figure 6, Supplementary Figures S4 and S7). Interestingly, the mutation of *Lhcb5* contributed to a more significant growth-defective phenotype when compared with *Lhcb6*. The phenotype differences in protonema and gametophyte between WT and mutants implied that Lhcb5 may function slightly differently from Lhcb6. Lacking both genes contributed to a decreased growth ratio in gametophyte, but not in protonema (Figure 6C, Supplementary Figure S5). Moreover, both mutants also showed disordered lamella arrangements of thylakoid, and abnormal extension of starch granules and plastoglobules in chloroplast, especially in *lhcb5* (Figure 3). Excess sunlight damages photosynthetic machinery and limits plant photosynthetic activity, growth, and productivity [39]. Spectroscopic characterization showed that the light-harvesting efficiency decreased more in mutants (Supplementary Figure S1). Under high-light stress, the NPQ of *lhcb6* and *lhcb5* decreased sharply, implying that the antenna proteins might participate in photo-protection by dissipating excess energy. Under abiotic stress, only *lhcb5* mutant showed any inhibition of NPQ, Y(II) and ETR, suggesting that the minor antenna protein, Lhcb5, played an important role in the excitation energy via non-photochemical pathways. The lower photo-inhibition PSII activity in *Lhcb*-defective mutants might connect with the limited growth rate and malformed phenotype in gametophyte. These results indicated that minor antenna proteins, Lhcb6 and Lhcb5, were involved in the gametophyte development in *Physcomitrella*. Light may be the critical factor in influencing *Physcomitrella* to diverge from seed and hydrophyte plants after land colonization.

In high-light conditions, Lhcb5 of Arabidopsis is activated in Zea-mediated photoprotection and catalyzed quenching of the qI type, while it is not phosphorylated in the state transition [14,22,23]. It has been reported that the deletion of *Lhcb5* does not cause alterations in several photosynthetic parameters, except in reduced growth [3,22,23]. In the present study, we found the minor antenna proteins Lhcb5 and Lhcb6 of *Physcomitrella* played an important role in the organization of photosynthetic complexes in grana partitions, participated in electron transport, and excess excitation energy dissipation under high-light stress in higher plants. Moreover, the loss of minor antenna protein Lhcb6 or Lhcb5 resulted in a pale-green leaf and the developmental defect phenotype, which has not been reported in higher plants or other green alga. Function analysis of minor antenna proteins, Lhcb6 and Lhcb5 in *P. patens,* provides evolutionary insights for the land plants.

## 4. Materials and Methods

### 4.1. Plant Materials and Treatments

The moss *P. patens* were cultured on modified BCD medium [41] in a growth chamber at 23 ± 1 °C with 16 h of light (55 μmol m^−2^ s^−1^) and 8 h of dark control light conditions. Uniform leafy shoots and protonema were obtained as described [42]. Two-week-old gametophore colonies were gently ground with a homogenizer, and transferred the homogenate to a cellophane overlay on solid BCD medium, containing 0.75% (*w*/*v*) agar, with 5 mM ammonium tartrate, and 0.5% (*w*/*v*) glucose. After a week, the cellophane overlay bearing regenerated protonema tissues was transferred onto ammonium tartrate-free BCD medium. The extended leaves and rhizoids were obtained for experimental analysis after three weeks. For light treatments, 5-day-old plants were transferred to 450 μmol m^−2^ s^−1^ for 3 h (HL) [20]. For osmotic treatments, the plants were transferred onto fresh agar plates of BCD medium containing 500 mM mannitol [43] for 1 day and 3 days, respectively (M1 and M3).

### 4.2. Protoplast Transformation and Mutant Identification

Genomic *P. patens* protonema DNA extraction was performed as previously described in [17], and used as a template for *PpLhcb5* (locus XM-001752760) and *PpLhcb6* (locus XM-001757486) cloning. Two homologous regions (upstream and downstream) of target coding sequences were amplified by PCR from the cDNA library of *P. patens* tissue and sub-cloned into pTN182 vector (Figure 1A) [44]. The transformation followed, as described previously in [41], with minor modifications. One-week-old protonemal tissues were collected for protoplast generation and PEG-mediated transformation. The selection and generation of resistant colonies were described previously [17]. The defects of *Lhcb5* and *Lhcb6* mutants were studied in three independent lines and were parallel. Quantitative PCR and immunoblotting showed that the genes or proteins were absent in both mutants. The primers used in this study were listed in Table S1.

### 4.3. Chlorophyll Content Measurement and EM Scanning

Leaf total chlorophyll was extracted with 80% acetone from two-week-old tissues and measured as previously described [22]. Samples from one-month-old tissues were fixed overnight in 3% glutaraldehyde and 0.1 M sodium cacodylate buffer (pH 6.9) at 4 °C. Ultrathin sections were cut with an ultramicrotome (Reichert-Jung, Ultracut, Wetzlar, Germany) and observed with a transmission electron microscope (FEI Tecnai G2 F20 S-TWIN, Hillsboro, OR, USA) operating at 75 kV [45].

### 4.4. Fluorescence Spectrum Analysis

The fluorescence emission spectrum was performed at room temperature using 436 nm excitation, 5 nm spectral bandwidth and 1 nm spectral resolution with a Hitachi F-4500 spectrofluorometer [46]. Samples were diluted at a chlorophyll concentration of 10 μg/mL in 20 mM Hepes/KOH, pH 7.8, 0.33 M sorbitol, 5 mM MgCl_2_ and 70% glycerol (*w*/*v*).

### 4.5. The Electron Transport Activity Analysis

The electron transport measurements were performed with a Clark type oxygen electrode (Hansatech) [47,48] at room temperature. The reaction mixture of whole chain electron transport activity contained reaction buffer: 1 mM methyl viologen (Sigma-Aldrich, St. Louis, MO, USA), 10 mM methylamine (Sigma-Aldrich), 1 mM NaN_3_ and thylakoid membranes. The reaction mixtures for PSII-catalyzed electron transport activity contained reaction buffer with 5 mM NH_4_C (Sigma-Aldrich), 5 μM DCPIP (Sigma-Aldrich) and thylakoid membranes. The PSI-catalyzed electron transport activity assay mixture contained 2 mM ascorbate, 100 mM DCPIP, 1 mM MV and 1 mM NaN_3_, 10 mM methylamine and 10 mM DCMU (Sigma-Aldrich). The thylakoid membranes concentration was 10 μg/mL.

### 4.6. Measurement of ΔpH

The kinetics of ΔpH formation across the thylakoid membrane were measured as previously described in [22]. The reaction buffer was as follows: 30 mM Tricine/NaOH, pH 7.8, 0.1 M sorbitol, 5 mM MgCl_2_, 10 mM NaCl, 20 mM KCl, 100 mM methyl viologen, and 2 mM 9-aminoacridine. The chlorophyll concentration in the reaction buffer was 30 μg/mL.

### 4.7. The Fluorescence and NPQ Measurement

The chlorophyll fluorescence was measured with a pulse-amplitude modulated chlorophyll fluorometer (mini-PAM) (Walz, Effeltrich, Germany) at room temperature, with saturating light of 6000 μmol·m^−2^·s^−1^ and actinic light of 830 μmol·m^−2^·s^−1^. For measurements, plants were dark-adapted for 30 min. Fm is the maximal fluorescence and F_0_ is the minimal fluorescence of dark-adapted leaves, Fm’ is the maximal fluorescence and F_0_′ is the minimal fluorescence of samples under light. The parameters Fv, Fv/Fm, Y(II), NPQ, qP, and relative ETR were calculated as Fm-F_0_, (Fm’-F_0_)/Fm’, (Fm’-F)/Fm’, (Fm-Fm’)/Fm’, (Fm’-F)/(Fm’-F_0_′) and Y(II)·PAR, respectively [33].

### 4.8. BN-PAGE and Immunoblotting Analysis

Thylakoids and functional chloroplasts were isolated from three-week-old protonemal tissue as previously described [49,50]. BN-PAGE was performed with slight modifications as described previously in [25]. Thylakoid was solubilized with 1% (*w*/*v*) n-dodecyl-β-d-maltoside (DM) and incubated on ice for 30 min. After centrifugation at 13,000× *g* for 10 min at 4 °C, the supernatant was supplemented with 0.1 vol sample buffer (100 mM BisTris/HCl, pH 7.0, 500 mM 6-amino-caproic acid, 30% (*w*/*v*) glycerol, 5% (*w*/*v*) Serva blue G) and subjected to BN-PAGE with a gradient of 5–13.5% acrylamide in the separation gel. The electrophoresis was performed at 4 °C, 125 V for 3–6 h.

Following Chen [9], isolated samples with 25 µg chlorophyll were loaded and electro-blotted on nitrocellulose membranes. The antibody of Lhca, Lhcb4, Lhcb5, and Lhcb6 (Agrisera, Sweden) were applied, and the signal was revealed by anti-rabbit IgG.

### 4.9. Measurement of Growth Rate

Analysis and identification of gametophyte and protonema tissue of *Physcomitrella* were photographed directly in their growth media as previously described in [51]. The live-image acquisition of protonema and gametophyte were performed with a light microscope (Nikon DS-Fi1c, Minato, Japan) and a digital camera (Leica DFC495, Wetzlar, Germany), respectively.

The growth-rate analysis of gametophyte in mutants and wild-type plants was carried out as previously described in [52]. The relative growth rate was estimated by measuring the fresh and dry weight of petri dish tissues for the same growth time.

### 4.10. Statistical Analysis

A statistical analysis of the results was carried out from experiments with three or more mean values using one-way analysis of variance (ANOVA). A difference was considered to be statistically significant when *p* ≤ 0.05 or very significant when *p* ≤ 0.01.

## Supplementary Materials

Supplementary materials can be found at https://www.mdpi.com/1422-0067/20/15/3665/s1. Figure S1: Spectroscopic characterization of wild-type and mutants; Figure S2: Measurement of trans-thylakoid ∆pH and the activity of the electron transport chain in wild-type and mutants; Figure S3: Time courses for induction and relaxation of NPQ of wild-type and mutants under 500 mM mannitol for 1 and 3 days; Figure S4: The protonema and gametophyte tissues of wild-type and mutants; Figure S5: The relative growth rate (RGR) of wild-type and mutants; Figure S6: Chlorophyll fluorescence parameters of WT and mutants; Figure S7: Micrographs of protonema (chloronema and caulonema) cells and gametophyte of WT and mutants; Table S1: Primers used in this study.
