# Supplementary material for: Involvement of Lhcb6 and Lhcb5 in Photosynthesis Regulation in Physcomitrella patens Response to Abiotic Stress
Source: Int J Mol Sci. 2019 Jul 26;20(15):3665. doi: 10.3390/ijms20153665 (PMC6695650; doi:10.3390/ijms20153665)
Supplement: Supplementary file 1 [file ijms-20-03665-s001.pdf]

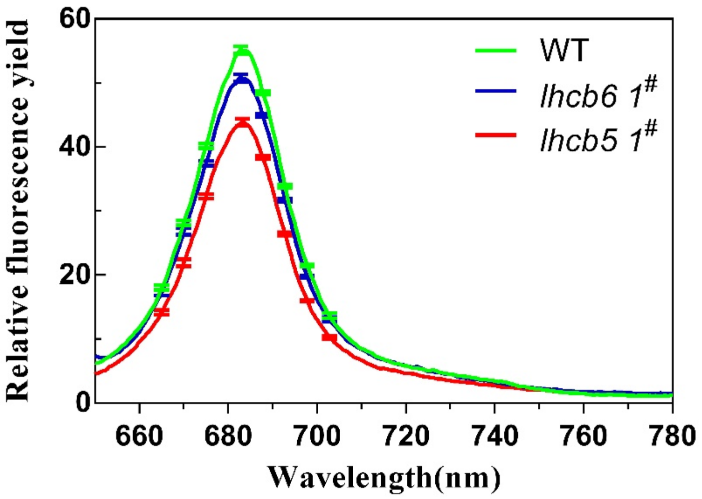

Supplementary Figure 1. Spectroscopic characterization of wild-type and mutants. The fluorescence emission spectra of thylakoid membranes were tested upon excitation 436 nm at room temperature. The data represent means ±SD of three biological replicates.

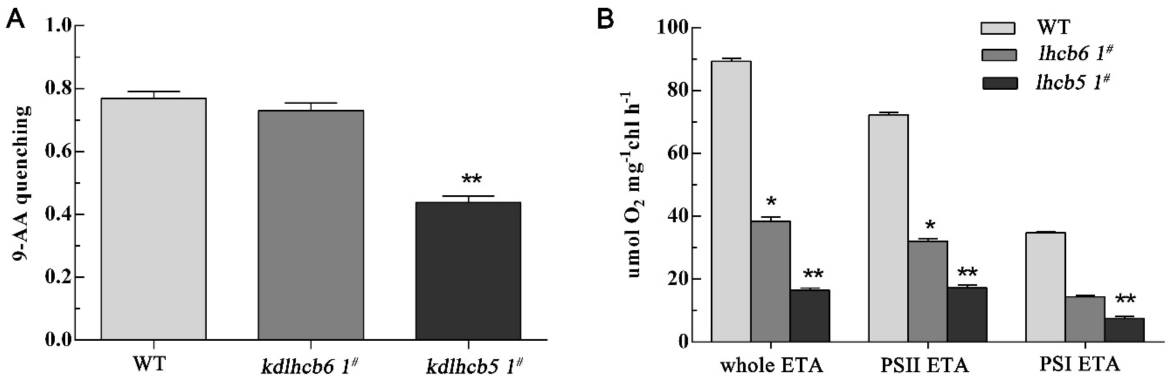

Supplementary Figure 2. Measurement of trans-thylakoid △pH and the activity of the electron transport chain in wild-type and mutants. (A) The fluorescence of 9-AA in intact chloroplasts was quantified as a measure for trans-thylakoid △pH. (B) The activity of the electron transport chain of mutants and wild-type thylakoids was mediate by indictor DCPIP. The data represent means ±SD of three biological replicates. Statistical significance compared with the wild-type p is indicated by asterisks (**P ≤ 0.01, *P ≤ 0.05, student's t-test).

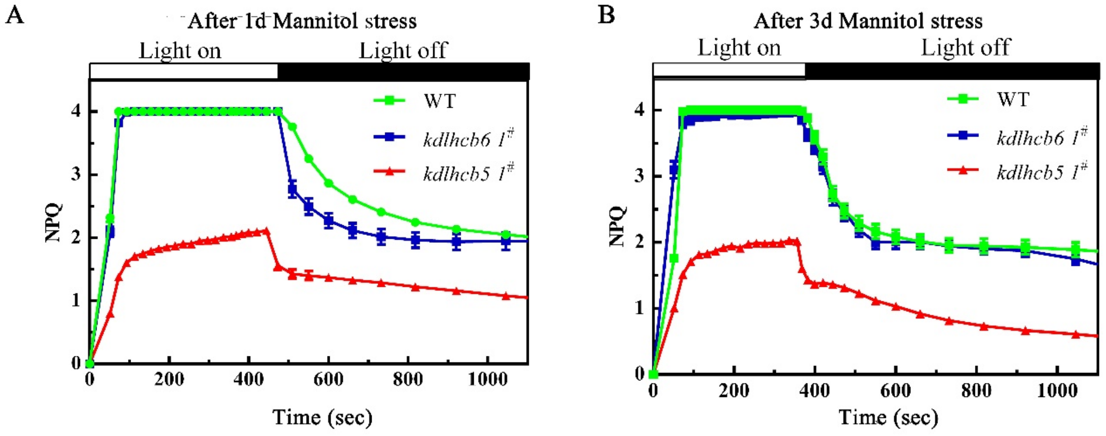

Supplementary Figure 3. Time courses for induction and relaxation of NPQ of wild-type and mutants under 500 mM mannitol for 1 and 3 days.

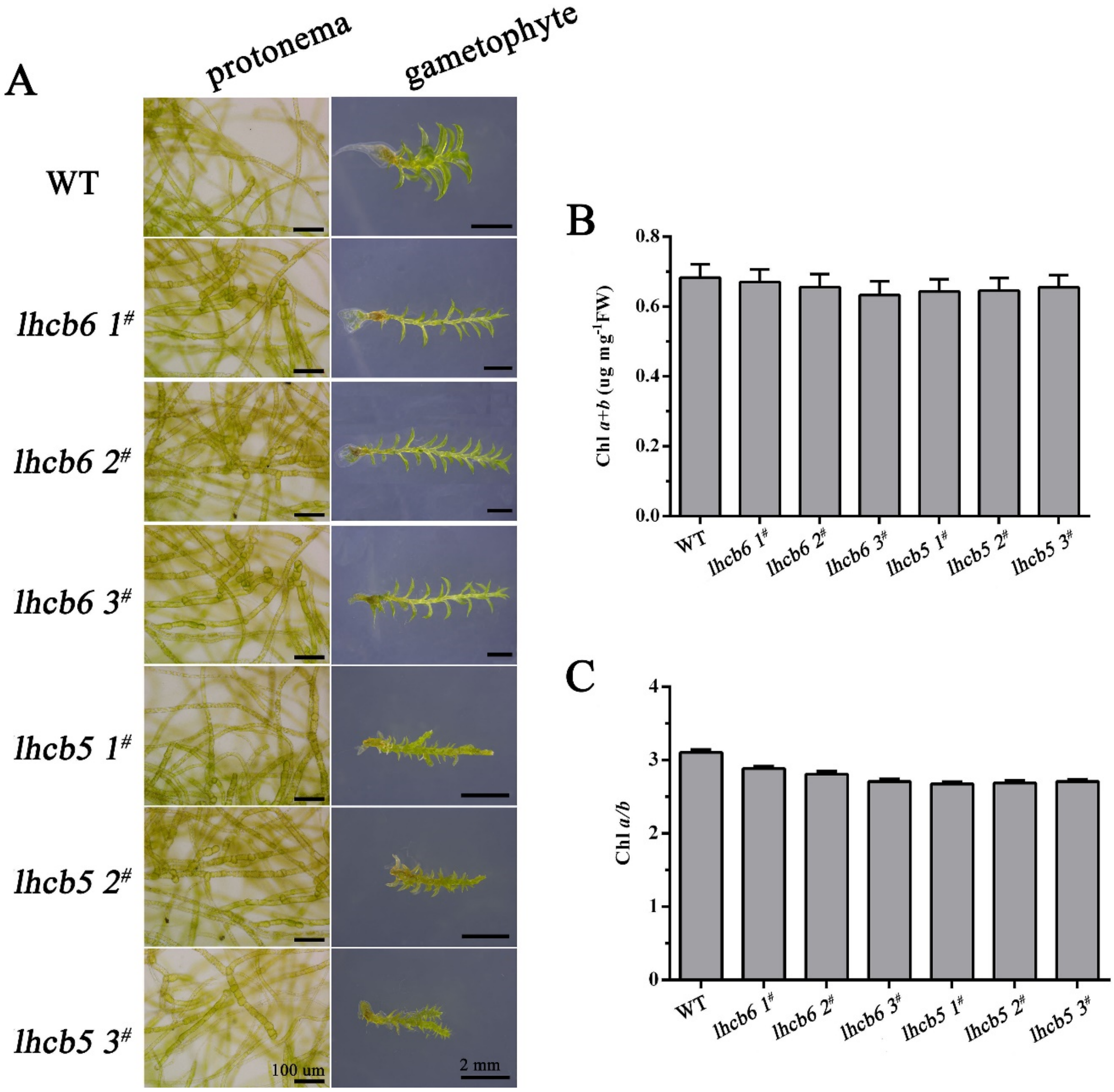

Supplementary Figure 4. The protonema and gametophyte tissues of wild-type and mutants. (A) Protonema filaments (left) and isolated leafy gametophyte (right) of *Physcomitrella*. The chlorophyll *a*+*b* content (B) and the ratio of chlorophyll *a*/*b* (C) of protonemal tissues in wild-type and mutants. FW, Fresh weight. The data represent the means ± SD of three biological replicates. Statistical significance compared with the wild-type is indicated by asterisks (**P ≤ 0.01, *P ≤ 0.05, student's t-test).

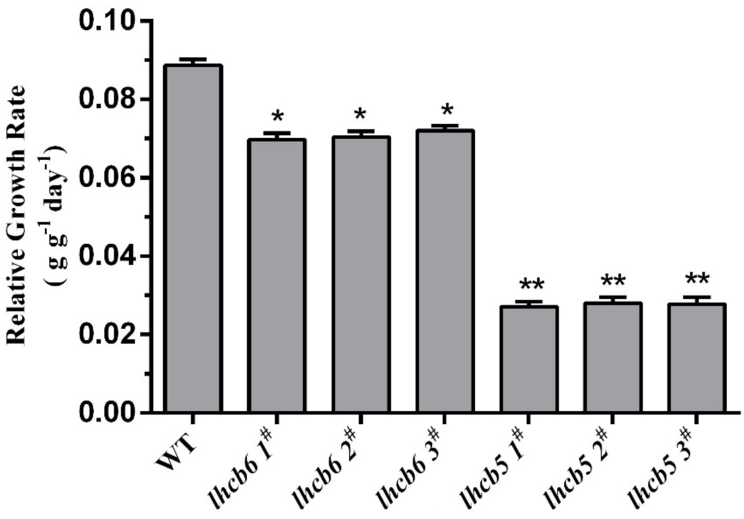

Supplementary Figure 5. The relative growth rate (RGR) of wild-type and mutants. The data represent the means ± SD of three biological replicates. Statistical significance compared with the wild-type p is indicated by asterisks (**P ≤ 0.01, *P ≤ 0.05, student's t-test).

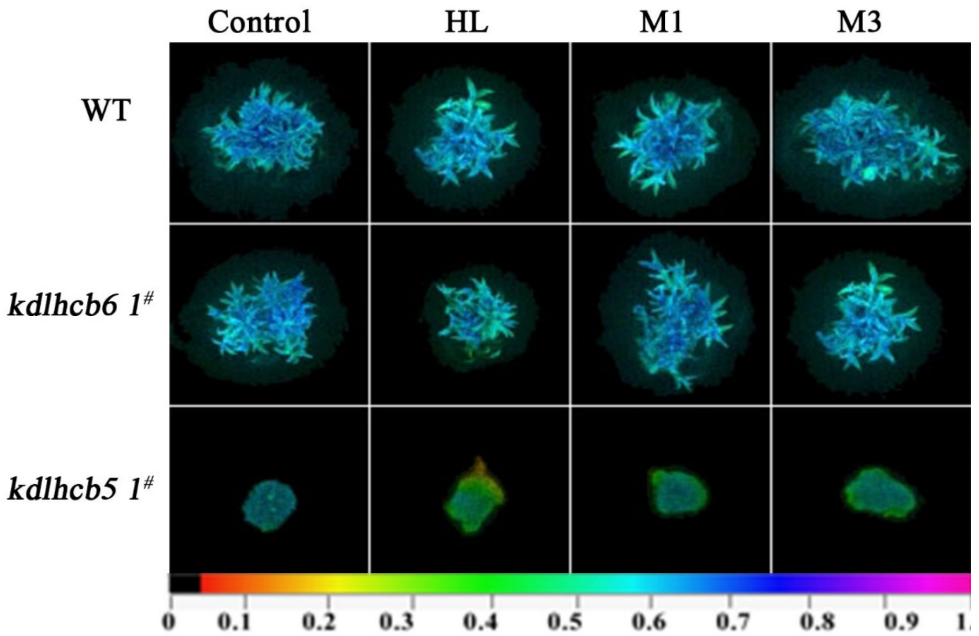

Supplementary Figure 6. Chlorophyll fluorescence parameters of WT and mutants. The false color code depicted at the bottom of the image ranged from 0 (black) to 1.0 (purple). HL, high light. M1 and M3, mannitol stress for 1 and 3 days, respectively. .

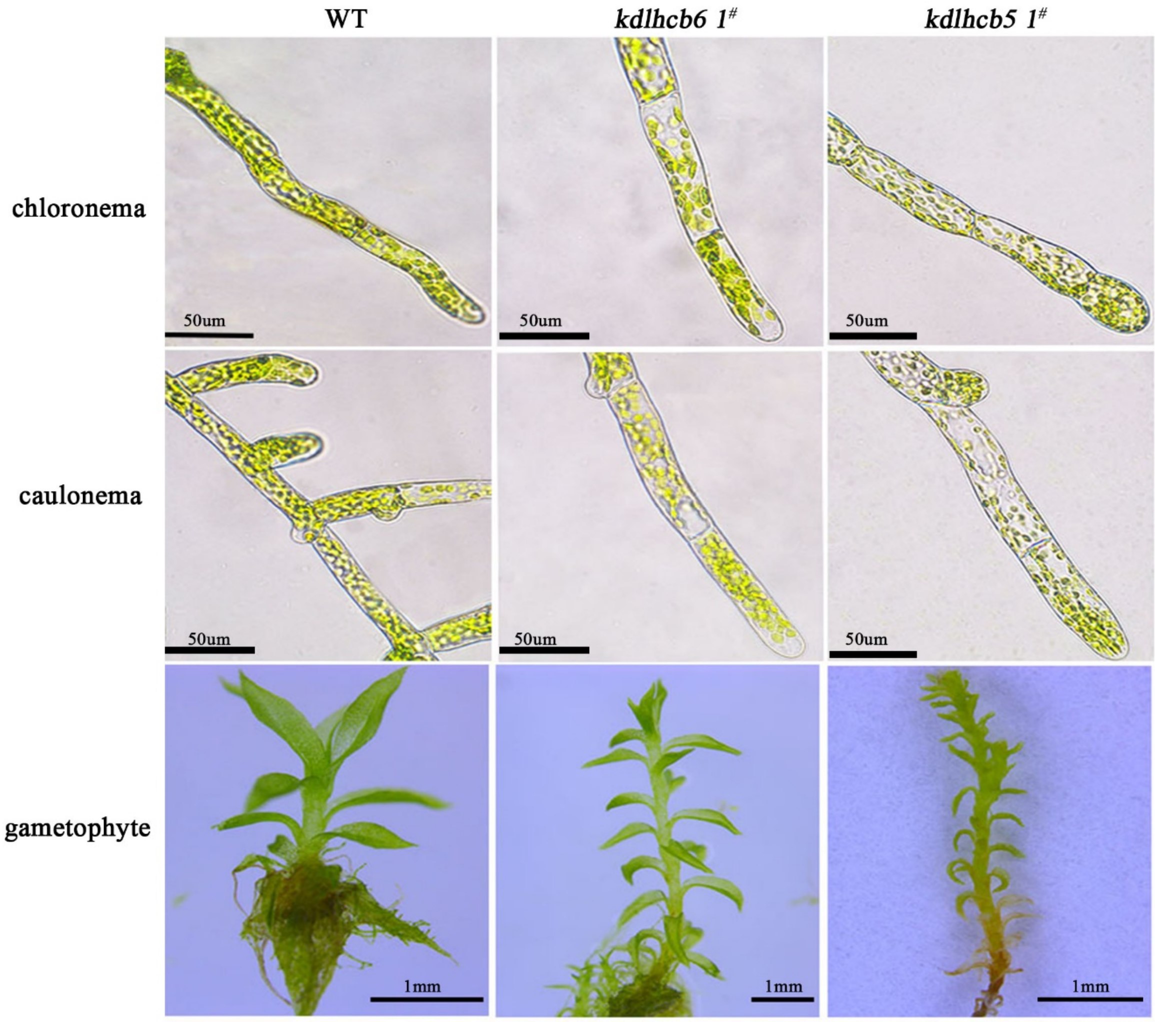

Supplementary Figure 7. Micrographs of protonema (chloronema and caulonema) cells and gametophyte of WT and mutants.

Table 1. Primers used in this study.

Gene	Primer name	Sequence (5′-3′)	Use
PpLhcb6	PpLhcb6 F-1#	ACGGGGTACCATGGCCACCAAGAAGGTGTCG	pTN182 construct
	PpLhcb6 R-1#	ACCCAAGCTTATGGAACGCCGCTCCATCCCT	pTN182 construct
	PpLhcb6 F-2#	CTAGTCTAGAGCAGTCGGTAGAGTGGGCAAC	pTN182 construct
	PpLhcb6 R-2#	ACGCGGATCCACAGACCCAAGGCACCGAGAG	pTN182 construct
	PpLhcb6 F-3#	CCGCCTTGAAATGGTATAGAGA	mutant screening
	PpLhcb6 R-3#	CTTGGAGAAGTCTGGCACGTAC	mutant screening
PpLhcb5	PpLhcb5 F-1#	ACGGGGTACCACTCAACGGAACTAAGGGAACA	pTN182 construct
	PpLhcb5 R-1#	ACCCAAGCTTAAGGCGGCTAAGGGTCTATG	pTN182 construct
	PpLhcb5 F-2#	CTAGTCTAGATTCGGATAAGGCGTAGCAGT	pTN182 construct
	PpLhcb5 R-2#	ACGCGGATCCGACCCACATTACATGAACAAGC	pTN182 construct
	PpLhcb5 F-3#	TCGCCGAGGTGATTCTG	mutant screening
	PpLhcb5 R-3#	TCTGCGGTTCCCTGGAT	mutant screening
PpLhcb4	PpLhcb4 F	ATGCGTCGCCCAGTTGT	RT-PCR
	PpLhcb4 R	ATGCGTCGCCCAGTTGT	RT-PCR
PpLhca3	PpLhca3 F	TGCCTCCAAGCAAAGCC	RT-PCR

	PpLhca3 R	CGCCATCATCGCCAACC	RT-PCR
PpLhcb9	PpLhcb9 F	ACGGGAAGTCAGTGGCA	RT-PCR
	PpLhcb9 R	CTGGGTCGCTTGAGAAT	RT-PCR
PpLhcsR	PpLhcsR 1F	TTGGCTCCCGTATTTCC	RT-PCR
	PpLhcsR 1R	TCGTCCCTCAAGGTGTT	RT-PCR
PpPsbS	PpPsbS F	CCTCGGCTTCGTTATTC	RT-PCR
	PpPsbS R	AGCACCCTTCACTCCCT	RT-PCR
Ppvde	PpVDE F	GGTGCTGGAAGCGTTAG	RT-PCR
	PpVDE R	GAGACCGAGGCAGTTGT	RT-PCR
PpEF1a	PpEF1a F	GCCAAGAAGAAGTGAATAGTGCG	RT-PCR
	PpEF1a R	ACGTCTGCCTCGCTCTAGC	RT-PCR
